# Interpreting Metabolomic Profiles using Unbiased Pathway Models

**DOI:** 10.1371/journal.pcbi.1000692

**Published:** 2010-02-26

**Authors:** Rahul C. Deo, Luke Hunter, Gregory D. Lewis, Guillaume Pare, Ramachandran S. Vasan, Daniel Chasman, Thomas J. Wang, Robert E. Gerszten, Frederick P. Roth

**Affiliations:** 1 Department of Biological Chemistry and Molecular Pharmacology, Harvard Medical School, Boston, Massachusetts, United States of America; 2 Cardiology Division, Massachusetts General Hospital, Boston, Massachusetts, United States of America; 3 Broad Institute of MIT and Harvard, Cambridge, Massachusetts, United States of America; 4 Center for Cardiovascular Disease Prevention, Brigham and Women's Hospital, Harvard Medical School, Boston, Massachusetts, United States of America; 5 Donald W. Reynolds Center for Cardiovascular Research, Brigham and Women's Hospital, Harvard Medical School, Boston, Massachusetts, United States of America; 6 Framingham Heart Study, National Heart, Lung, and Blood Institute and Boston University, Boston, Massachusetts, United States of America; 7 Sections of Cardiology and Preventive Medicine, and the Whitaker Cardiovascular Institute, Boston University School of Medicine, Boston, Massachusetts, United States of America; 8 Center for Immunology and Inflammatory Diseases, Massachusetts General Hospital, Boston, Massachusetts, United States of America; University of California, San Diego, United States of America

## Abstract

Human disease is heterogeneous, with similar disease phenotypes resulting from distinct combinations of genetic and environmental factors. Small-molecule profiling can address disease heterogeneity by evaluating the underlying biologic state of individuals through non-invasive interrogation of plasma metabolite levels. We analyzed metabolite profiles from an oral glucose tolerance test (OGTT) in 50 individuals, 25 with normal (NGT) and 25 with impaired glucose tolerance (IGT). Our focus was to elucidate underlying biologic processes. Although we initially found little overlap between changed metabolites and preconceived definitions of metabolic pathways, the use of unbiased network approaches identified significant concerted changes. Specifically, we derived a metabolic network with edges drawn between reactant and product nodes in individual reactions and between all substrates of individual enzymes and transporters. We searched for “active modules”—regions of the metabolic network enriched for changes in metabolite levels. Active modules identified relationships among changed metabolites and highlighted the importance of specific solute carriers in metabolite profiles. Furthermore, hierarchical clustering and principal component analysis demonstrated that changed metabolites in OGTT naturally grouped according to the activities of the System A and L amino acid transporters, the osmolyte carrier SLC6A12, and the mitochondrial aspartate-glutamate transporter SLC25A13. Comparison between NGT and IGT groups supported blunted glucose- and/or insulin-stimulated activities in the IGT group. Using unbiased pathway models, we offer evidence supporting the important role of solute carriers in the physiologic response to glucose challenge and conclude that carrier activities are reflected in individual metabolite profiles of perturbation experiments. Given the involvement of transporters in human disease, metabolite profiling may contribute to improved disease classification via the interrogation of specific transporter activities.

## Author Summary

Human disease is complex, arising from the interaction of many genetic and environmental factors. Efforts to personalize treatment have been thwarted by “phenotypic heterogeneity”, the apparent similarity of disease states with diverse underlying causes. One approach to resolve this heterogeneity is to redefine diseases on the basis of abnormal physiologic activities, which should allow grouping patients into categories with similar treatment response and prognosis. Physiologic activities can be identified and assessed through quantitative measurements of biomolecules—proteins, mRNAs, metabolites—in individual patient samples. The field of metabolomics involves the analysis of a broad array of metabolite levels from clinical fluid samples such as blood or urine and can be used to evaluate disease states. Because metabolic profiles are complex, we have taken an integrative network-based approach to understand them in terms of abnormal activities of enzymes and small molecule transporters. We have focused on the oral glucose tolerance test, used to diagnose diabetes, and have found that multiple transporters play an important role in the normal response to ingesting sugar. Many of these transporter activities are abnormal in individuals with impaired glucose tolerance and differing activities among them may reflect the diverse underlying causes and variable clinical courses of such patients.

## Introduction

Disease heterogeneity has challenged the practice of medicine. Individuals with the same apparent disease at our current diagnostic resolution often show remarkable variation in prognosis and treatment responsiveness, presumably because a superficially similar disease state can arise from diverse combinations of genetic and environmental factors [1]. Efforts to resolve the heterogeneity have focused on collecting increasing amounts of quantitative patient information, including genotypic [2] and mRNA [3] and protein expression data [4] with the hope of establishing better clinical classifiers based on aberrant activities of specific, targetable biological pathways.

Using tumor biopsy samples, oncologists are now exploring the incorporation of genomewide expression profiling into therapy [5],[6]. However, for complex human diseases that span multiple organ systems, metabolomics—the analysis of a broad array of metabolite levels from biologic fluid samples such as blood or urine—represents a minimally-invasive way to obtain quantitative biologic information from patients to uncover disease pathophysiology and aid diagnostic and prognostic classification [7].

Metabolomics data analysis may be facilitated by techniques applied to other high-throughput ‘omic data types. For microarray data, the integration of network information from protein-protein interaction data or predefined biologic pathways has greatly assisted elucidation of underlying processes and led to the development of increasingly robust and accurate gene-based classifiers for disease [8],[9]. We hypothesize that the characterization of human disease by metabolomic profiling should similarly benefit from interpreting metabolite changes in the context of known metabolic reactions.

We use data derived from oral glucose tolerance tests (OGTT) in 25 individuals with normal (NGT) and 25 with impaired (IGT) glucose tolerance [10]. We first sought significant overlaps between observed metabolite changes and preconceived definitions of metabolic pathways. Next we applied an unbiased pathway analysis by mapping the metabolite changes to a recent reconstruction of the human metabolic network [11] and use a recently developed variant [12] of previous approaches [13] derived for mRNA expression analysis to find active metabolic modules—connected subnetworks of highly changed metabolites. While the biased approach yielded little, the resulting unbiased pathway models highlight the interconnectedness between changed metabolites and propose a role for solute carriers in OGTT metabolite profiles. Hierarchical clustering and principal component analysis confirmed the importance of specific transporters by demonstrating that metabolites cluster naturally according to activities of the System A and L amino acid and SLC6A12 osmolyte transporters. Furthermore, they suggest an important role for the SLC25A13 mitochondrial aspartate-glutamate transporter in interindividual metabolite profile variability. Comparison of NGT and IGT active modules suggest blunted glucose- and/or insulin-stimulated enzyme and transporter activities in the IGT group. Given that transporters are implicated in multiple human diseases, the interrogation of transporter activities by perturbation-based metabolic profiling may ultimately contribute to improved disease classification and resolution of disease heterogeneity.

## Results

### Predefined Pathways Show Little Enrichment for Changed Metabolites

We examined metabolite profiles from a previously descibed oral glucose tolerance experiment (OGTT) [10], which involved the use of metabolite profiling to monitor physiologic responses to oral glucose challenges in individuals with normal (NGT) and impaired glucose tolerance (IGT). Multiple metabolites were changed significantly in response to glucose in two separate NGT populations. Furthermore, interpreting the list of changed metabolites in terms of known mechanisms of insulin action allowed the authors to assign the observed results to established biochemical pathways, including glycolysis, lipolysis and ketogenesis, and led to the proposal of new downstream pathways of insulin action, such as bile acid metabolism [10]. Many of the changed metabolites were not, however, mapped to established pathways.

We were thus interested in further elucidating the underlying biologic processes leading to the observed pattern of changes. Analyzing the OGTT metabolite profiles of the 25 NGT and 25 IGT Framingham Heart Study participants (see Methods), we identified 57 and 31 metabolites, respectively, changed at an FDR of 0.05 (see Table S1). We first revisited whether the pattern of changed metabolites was consistent with predefined metabolic pathways using the FuncAssociate program [14]. FuncAssociate uses a hypergeometric test and correction for multiple hypothesis testing to formally evaluate statistical significance for pathway enrichment (see Methods). Although originally designed to identify enriched “gene sets” among a list of genes, FuncAssociate can be adapted for “metabolite sets”. We used a recent reconstruction of the human metabolome, “Recon 1”, as a source of pathway information [11]. The significantly changed metabolites were ranked by magnitude of change and FuncAssociate was used to identify significant enrichment of any of the 99 separate metabolic pathways in Recon 1.

We evaluated NGT and IGT individually (comparing metabolite abundance before and after oral glucose load) and found enrichment solely in NGT for Bile Acid Biosynthesis at an adjusted *p*-value <0.001.

### Active Module Analysis Elucidates Metabolic Pathways of OGTT

The low yield of pathway enrichment could arise in part from the sparseness of our metabolome coverage or from the fact that most metabolites are implicated in multiple pathways. Furthermore, even if a pathway has uniformly increased flux, this will not generally lead to uniform increases in metabolite abundance. The relationship between enzymatic activity and metabolite concentration can be understood in terms of the relative contribution of “metabolic regulation” and “hierarchical regulation”. Metabolic regulation involves control of reaction flux through the interaction of enzymes with the rest of the metabolic network, such as changing substrate, product or modifier concentrations [15]. On the other hand, hierarchical regulation achieves control through changes in maximal enzyme activity, typically by altered gene expression. In the extreme case where there is simultaneous and proportional modulation of the activity of all enzymes in the pathway, one would see no changes in metabolite concentrations in a pathway despite changes in metabolic flux. A final explanation for the low yield of enriched predefined pathways may be that the physiologic perturbation only affects a subnetwork of metabolites that may not correspond to any of the preconceived pathway definitions. In light of these possibilities, we investigated the application of additional, emerging bioinformatics approaches, which emphasize unbiased pathway models.

We based our analysis on the fact that metabolites are linked via chemical reactions. We hypothesized that OGTT is a physiologic stimulus that alters flux through specific metabolic reactions. Since products from one reaction may serve as reactants for and drive other reactions, we sought groups of metabolites that are connected through metabolic reactions and collectively show a high degree of change. Furthermore we hypothesized that a perturbation such as OGTT would increase the activity of enzymes and transporters, many of which have multiple substrates. Thus, we were also interested in groups of changed metabolites linked by virtue of being substrates of a common enzyme or transporter.

We framed the search for functionally-linked, highly changed metabolites in OGTT in terms of the discovery of active modules (or subnetworks). Active module approaches have previously been applied in bioinformatics analysis to elucidate underlying biologic processes in gene expression data. In such analyses, the investigators typically overlaid gene scores based on differential expression in microarray experiments onto protein-protein interaction [16],[17] and/or transcription-regulatory [13] networks and looked for highly-connected, differentially expressed genes. We undertook a similar approach, combining OGTT metabolite profiles with metabolic reaction information.

**Figure 1:**
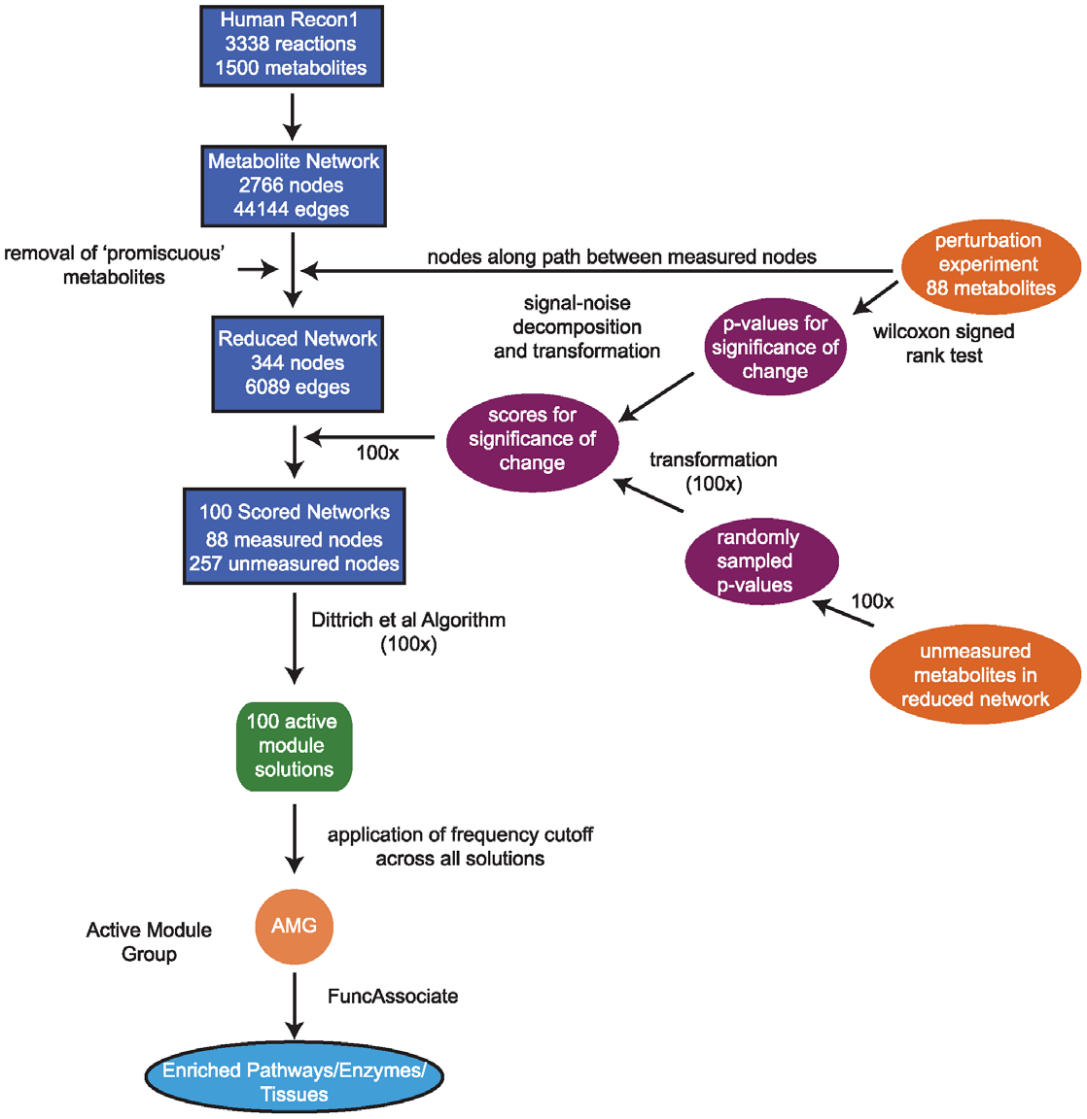
Analysis flowchart for metabolic reaction network construction, active module discovery, and evaluation of active module sets for enrichment for predefined biologic pathways, enzymes/transporters, and tissue activity.

We first built a Metabolic Reaction Network (MRN) using the 3338 metabolic reactions in Recon 1. Although Recon 1 includes most known transport reactions, the specific transporters were not always explicitly mentioned. Thus we expanded this list with 737 additional reactions explicitly modeling transport processes for the metabolites measured in this experiment (see Methods, Table S2), highlighting the relevant transporter for each reaction. We treated all reactants and product metabolites as nodes. Cellular locations were assigned to each metabolite as specified in Recon 1, and metabolites were split into multiple nodes (each corresponding to a different location). For example, five nodes in the MRN were assigned to D-Glucose, corresponding to glucose in the cytoplasmic, lysosomal, Golgi, endoplasmic reticulum and extracellular compartments. Edges were drawn between reactants and products in chemical reactions (see Methods and Figure 1) and between all substrates for each of the known enzymes or transporters catalyzing metabolic reactions (Table S2). In effect, we proceeded from a bipartite undirected graph [18], where both metabolites and proteins (enzymes/transporters) are represented as nodes, and interactions between metabolites and proteins represented as edges, to a unipartite metabolite interaction graph, where metabolites that are common substrates of enzymes or transporters were connected by edges. For those reactions where enzymes/transporters are unknown or unneeded, the corresponding reactant and product metabolites were directly connected.

We converted experimental measures of significance of change (*p*-values) for metabolites to scores (see Methods). Since Recon 1 includes cellular locations for reactions, if a metabolite had multiple cellular locations, different active modules could emerge, depending on the location to which a score was assigned. We hypothesized that the plasma metabolite profiles of perturbation experiments reflect altered flux in both intracellular reactions and in metabolite transport between the cell and plasma. We focused on each of these processes separately, building two scored MRNs. In the first, the Scored Extracellular MRN (EMRN), we assigned the score to the extracellular metabolite, modeling extracellular levels as being reflected in plasma. We also built a separate Scored Cytoplasmic MRN (CMRN), assigning the same scores to the cytoplasmic metabolite to better capture interconnections via intracellular reaction processes (we thus make the assumption that extracellular transport is not limiting, and that plasma metabolite abundance reflect intracellular abundances). We then looked for active modules, which represent connected subnetworks with high aggregate activity, using a recently published algorithm, developed for mRNA expression analysis [12], to provide an exact solution. We pursued active module searches for NGT and IGT, for both the EMRN and CMRN. Since not all metabolites in the MRNs were measured in the metabolomics experiment, we randomly sampled scores for the remaining metabolites and computed 100 active module solutions for each of the scored MRNs (see Methods, Figure 1).

Distributions of active module scores were evaluated for statistical significance relative to those obtained from random networks, where metabolite scores were permuted randomly amongst measured nodes. At an FDR threshold of 0.01, all of the solutions were highly significant (*p* = 8.5×10^−8^ for NGT-EMRN, 7.4×10^−9^ for NGT-CMRN, *p* = 7.0×10^−15^ for IGT-EMRN, *p* = 0.025 for IGT-CMRN, respectively, Mann-Whitney-Wilcoxon test) indicating that the clustering of metabolite changes in the network is highly non-random.

### Characterizing Active Modules of OGTT using FuncAssociate

We selected all metabolites that appeared with sufficient frequency (see Methods) across the active module solutions. This resulted in an Active Module Group (AMG) for each of NGT-EMRN, NGT-CMRN, IGT-EMRN, and IGT-CMRN. Metabolite frequencies for searches are shown in Table S3. As the AMGs represent unbiased pathway models for the OGTT experiment, we sought to characterize their relationship to predefined biological processes. We first repeated the FuncAssociate analysis and found marginal enrichment for Glycerophospholipid Metabolism (*p*_adj_ = 0.029) for the NGT-EMRN AMG and more convincing enrichment for Glycine, Serine, and Threonine Metabolism (*p*_adj_ = 0.008) within the NGT-CMRN AMG. However, these enriched predefined pathways encompass very few of the AMG metabolites.

We next sought to characterize whether the AMG metabolites are active in any particular human tissue. To do so, we exploited recent predictions of which metabolic reactions in the Recon 1 network were likely to be active in ten specific human tissues, using constraint-based flux modeling [19]. We tested whether AMGs correspond to predicted metabolic activities in any of these tissues (see Methods). AMGs all showed enrichment (*p*_adj_<0.05) for metabolites predicted to be active in kidney and/or liver, suggest that OGTT responses primarily involve metabolites produced in and/or consumed in these organs. Both tissues are established targets of insulin action, with liver demonstrating increased glycogen storage and fatty acid production and decreased gluconeogenesis [20] and kidney showing changes in electrolye clearance [21] in response to insulin.

### Active Modules Suggest Amino Acid Transporters Involved in Glucose Response

**Figure 2:**
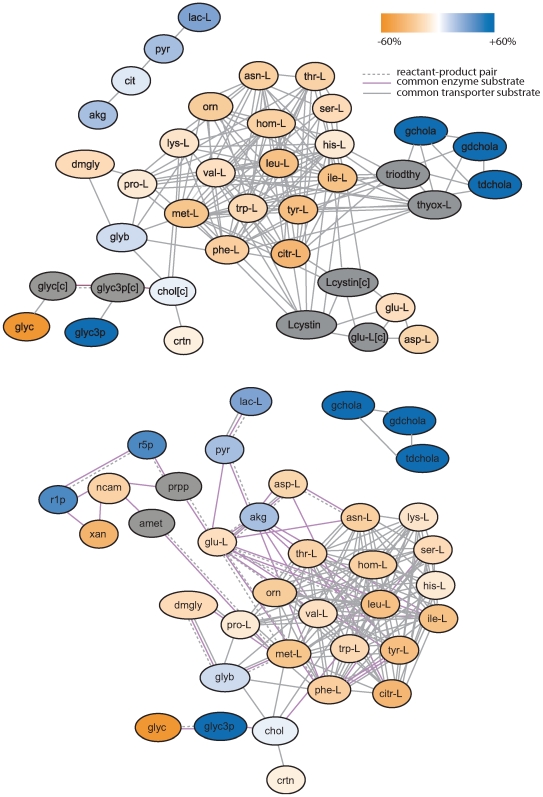
Active Module Groups from the NGT-EMRN and NGT-CMRN. Panels (a) and (b) correspond to NGT-EMRN and NGT-CMRN, respectively. Nodes in the AMGs correspond to metabolites in chemical reactions and edges are drawn between reactant-product pairs or shared substrates of enzymes/transporters. A gradient from gold to blue was used to denote reduced percentage change in metabolite abundance after glucose challenge. For clarity, changes were truncated at ±60%. Unmeasured nodes are shown in grey. Edges corresponding to different types of functional links between metabolites are indicated. Cellular locations for metabolites in (a) are assumed to be extracellular unless denoted by [c] for cytoplasmic. Likewise, cellular locations in (b) are assumed to be cytoplasmic unless denoted by [e] for extracellular. The lac-pyr-cit-akg group of metabolites in (a) is connected to the remainder of the set via metabolites with relative frequencies<0.20 across solutions; the same is true of the bile salts cluster in (b).

An inspection of the AMGs for NGT samples (Figure 2) revealed a central cluster of highly interconnected standard (15) and nonstandard amino acids (5), 19 of which decrease in plasma in response to glucose challenge. (The AMGs for IGT samples (Figure S1) consist entirely of standard (11) and non-standard amino acids (2) and are discussed further below.) Standard amino acids represent building blocks for proteins, whereas nonstandard amino acids such as citrulline, ornithine, dimethylglycine and homoserine are implicated in other biologic processes (see below). Within the EMRN, all edges between amino acids correspond to shared transporters, while within the CMRN, in addition to shared transporter activity edges, some edges constitute reactant-product pairs and/or shared enzyme substrates. The interconnectedness of both standard and nonstandard changed amino acids in the AMG supports the hypothesis that metabolite profiles reflect both increased protein synthesis and altered amino acid transporter activity. In fact, it is known that certain amino acid transporter activities are activated in response to insulin (see below).

Amino acid transport activities have historically been grouped into “Systems” that describe the chemical properties of the transported molecules (e.g. cationic or small/neutral) and the response to specific inhibitors [22]. Transporters can also be classified by whether their transport activity is primarily effected via facilitated diffusion or exchange reactions and also by the co-transported ions (e.g. sodium, protons, potassium, or chloride). Individual transporters, once cloned, have been mapped to these Systems. We used FuncAssociate to identify which enzymatic activities or transporters are overrepresented in the AMGs. Table 1 indicates enzymes or transporters with substrate profiles demonstrating significant overlap with AMG metabolites in the experiment. Transporters with broad amino acid specificity such as the SLC6 and SLC7 families are favored in the FuncAssociate enrichment analysis. However, given that many of these transporters have tissue-specific activities, there is probable involvement of other transporter families in glucose-stimulated amino acid influx, including the SLC38 (SNAT) family of small polar amino acid transporters, and the SLC1 family of anionic amino acid transporters. The SLC38A2 transporter, a weakly-accumulating neutral amino acid transporter, is in fact known to be post-translationally regulated by insulin in murine adipocyte [23] and rat skeletal muscle cell lines [24]. Furthermore, insulin has been shown to increase System A (small, neutral amino acid) and System L (large hydrophobic) amino acid transport activities in cultured trophoblasts [25]. A complete list of all possible transporters and enzymes corresponding to the edges in the AMGs is provided in Table S4.

**Table 1:** Enrichment for enzymes and transporters in the NGT and IGT active module groups.

Enzyme or Transporter Family	Enzyme or Transporter Family Member	System	Measured Substrates in Active Module Groups	Reaction	Tissue Distribution
SLC6	SLC6A14*	B(0,+)	Citr-L, Leu-L, Ile-L, Met-L, Lys-L, Val-L, Phe-L, Tyr-L, Trp-L, His-L, Orn-L, Ser-L, (reduced transport for Thr-L, Hom-L, Asn-L, Gln-L)	Facilitated	lung, trachea, salivary gland, mammary gland, pituitary, stomach, colon
SLC6	SLC6A15†	NA	Val-L, Leu-L, Met-L, Ile-L	Facilitated	brain
SLC6	SLC6A19*	B(0)	Citr-L, Leu-L, Ile-L, Phe-L, Trp-L, Tyr-L, Gln-L, Met-L, Asn-L, Hom-L, Thr-L, Ser-L	Facilitated	kidney, intestine
SLC3/SLC7	SLC7A1†	y+	Lys-L, Arg-L, Orn-L, His-L	Facilitated	Ubiquitous except liver
SLC3/SLC7	SLC7A2†	y+	Lys-L, Arg-L, Orn-L, His-L	Facilitated	liver, skeletal muscle, pancreas
SLC3/SLC7	SLC7A3†	y+	Lys-L, Arg-L, Orn-L, His-L	Facilitated	thymus, overy, testis, brain
SLC3/SLC7	SLC3A2/SLC7A5*	L	Tyr-L, Phe-L, Trp-L, Leu-L, Ile-L, Val-L, His-L, Citr-L	Exchange	brain, ovary, testis, placenta
SLC3/SLC7	SLC3A2/SLC7A8†	L	Citr-L, Gln-L, Leu-L, Ile-L, Met-L, Val-L, Phe-L, Thr-L, Asn-L, Trp-L, Ser-L, Tyr-L, Hom-L	Exchange	kidney, intestine, brain, placenta, ovary, testis, muscle, epithelium
SLC3/SLC7	SLC3A1/SLC7A9*	b(0,+)	Lys-L, Val-L, Orn-L, Met-L, Ile-L, Leu-L	Exchange	kidney, intestine, lung, placenta, brain, liver, endothelium
SLC43	SLC43A1*	L	Val-L, Ile-L, Citr-L, Leu-L, Phe-L, Met-L	Facilitated	kidney
SLC43	SLC43A2*	L	Val-L, Ile-L, Citr-L, Leu-L, Phe-L, Met-L	Facilitated	kidney
SLC38	SLC38A4†	A	Met-L, Lys-L, His-L, Arg-L, Asn-L, Ser-L	Facilitated	liver, skeletal muscle, kidney, pancreas
SLCO1	SLCO1A2†	NA	taurochenodeoxycholate, glycocholate, glycochenodeoxycholate	Facilitated	brain, kidney, liver, ciliary body
SLCO1	SLCO1B1†	NA	taurochenodeoxycholate, glycocholate, glycochenodeoxycholate	Facilitated	liver
SLCO1	SLCO1B3†	NA	taurochenodeoxycholate, glycocholate, glycochenodeoxycholate	Facilitated	liver

The FuncAssociate program [14] was used to identify enzymes and transporters that contributed a greater number of metabolites to the AMGs than expected by chance. Amino acid transporters with substrate profiles overlapping AMG metabolites organized in groups, with the transport system, AMG. substrates and mode of reaction (facilitated diffusion vs. exchange) and tissue distribution are indicated [22].

*Transporters/enzymes with *p_adj_*<0.0125 (p<0.05 with Bonferonni correction for 4 AMGs experiments tested for enrichment).

†transporters with *p_adj_* = 0.0125–0.05.

### Active Modules Identify the Interconnectedness of Multiple Changed Metabolites

In addition to the core cluster of amino acids, the AMGs include additional changed metabolites on their periphery. These peripheral metabolites are connected to the amino acid core via unmeasured metabolites, which represent potential functional links. For example, in the NGT-CMRN AMG (Figure 2B), glutamate is linked to nicotinamide and ribose-5-phosphate via the unmeasured metabolite phosphoribosyldiphosphate (PRPP). Glutamate and PRPP are a reactant-product pair and common substrates of the enzyme glutamate-PRPP amidotransferase. In the corresponding reaction, involved in purine biosynthesis, glutamine transfers an amine group to PRPP to form glutamate and ribosylamine-5-phosphate. Ribosylamine-5-phosphate, in turn, is a building block for *de novo* purine biosynthesis. Interestingly, many of the other peripheral changed metabolites linked to PRPP in the NGT-CMRN AMG are also involved in nucleotide biosynthesis. Ribose-5-phosphate, which is interconverted with ribose-1-phosphate (R1P; the two cannot be distinguished on our mass spectrometry platform), is phosphorylated to form PRPP. R1P combines with xanthine (and hypoxanthine) as part of the purine salvage pathway of nucleic acid biosynthesis, in a reaction catalyzed by purine nucleotide phosphorylase (PNP). PNP also catalyzes the reaction of nicotinamide with R1P to ultimately form nicotinamide adenine dinucleotide (NAD). Thus the peripheral metabolite cluster shown in NGT-CMRN captures the interrelationship of the various metabolites involved in insulin-stimulated purine nucleotide biosynthesis [26].

The other peripheral metabolite clusters in the NGT-CMRN and NGT-EMRN AMGs capture other insulin-stimulated activities including glycolysis, triglyceride biosynthesis, and an increase in bile salt plasma levels (by unknown mechanisms). Although these were commented upon previously [10], we note that the bile salts are linked by edges corresponding to common transporters (see Figure 2, Table 1)–thus one mechanism not noted previously by which glucose/insulin could increase plasma bile salt levels is via increased transporter activity, with diffusion outwards along a concentration gradient.

### A Role for the Osmoregulatory Transporter SLC6A12 in the Glucose Response

**Figure 3:**
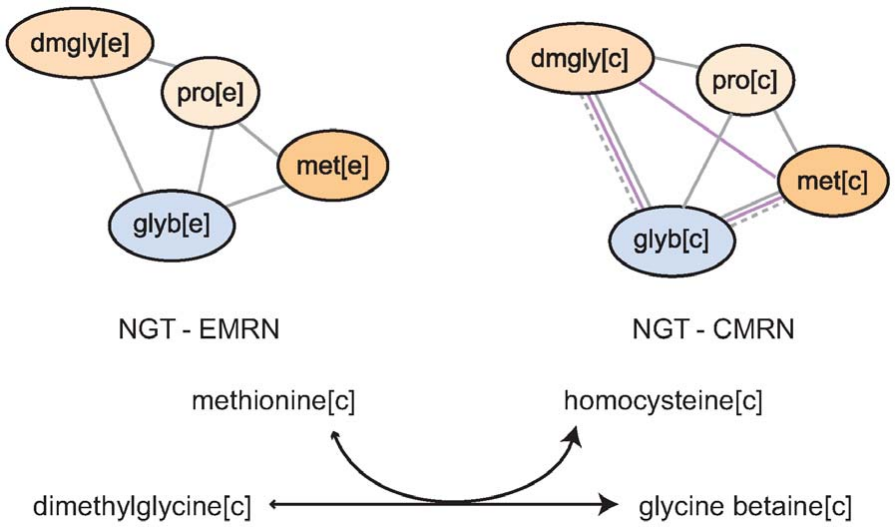
Proposed mechanism for coupling of methionine influx to SLC6A12 transport of glycine betaine and dimethylglycine for osmoregulation. The connections among the 3 metabolites (and proline) in the NGT-EMRN and NGT-CMRN AMGs are shown, along with the Recon 1 betaine-homocysteine methyltransferase catalyzed reaction.

For the NGT group, there is a significant drop in L-Proline and N,N-dimethylglycine levels and an increase in glycine betaine levels. All three amino acids appear in the NGT-EMRN and NGT-CMRN AMGs with the edges between them representing shared transport by the SLC6A12 carrier [27] (Figure 2). SLC6A12 is an ancient, highly-conserved osmoregulator, which controls cellular volume by regulating extrusion of the osmolytes GABA and glycine betaine when placed in solutions of varying osmolarity [28]. SLC6A12 can also transport proline, diaminobuytric acid, and beta-alanine, and to a lesser extent glycine, putrescine, dimethylglycine and choline [29]. Interestingly, from the CMRN we see that glycine betaine and dimethyl glycine are also reactants and products in a metabolic reaction (catalyzed by betaine-homocysteine methyltransferase), which involves a reversible transfer of a methyl group from betaine to homocysteine, resulting in methionine and dimethylglycine (Figure 3). Insulin-triggered amino acid influx could, in fact, be coupled to glycine betaine extrusion since methionine influx would tend to drive the betaine-homocysteine reaction in reverse, leading to depletion of cellular dimethylglycine and increased glycine betaine. The latter two metabolites could then follow their respective concentration gradients resulting in betaine export and dimethylglycine import, explaining the respective increase and decrease in plasma levels. Presumably, the purpose of this coupling is to maintain cell osmolarity in face of the amino acid/glucose influx brought about by insulin.

### Comparison of NGT and IGT Active Modules

The IGT-EMRN and IGT-CMRN (Figure S1) consist exclusively of a group of amino acids with decreased plasma level upon glucose load. Neither includes the SLC6A12 substrates, bile salts, and citric acid cycle metabolites, glycerol or glycerol-3-phosphate, or the purine nucleotide metabolism substrates. Thus, the glucose- and/or insulin-stimulated changes in the corresponding enzyme or transporter activities appear to have been blunted in the IGT group.

### Changed Metabolites Cluster According to Transporter Activity

Although the AMGs convincingly illustrate that changed metabolites are common substrates of small molecule transporters, they cannot establish coordinated activity of these cotransported substrates. To explore whether metabolite substrates of individual transporters are in fact coregulated, we performed hierarchical clustering across the 25 individuals from the NGT and IGT groups, looking to identify metabolites that show a similar absolute percentage of change across individuals.

**Figure 4:**
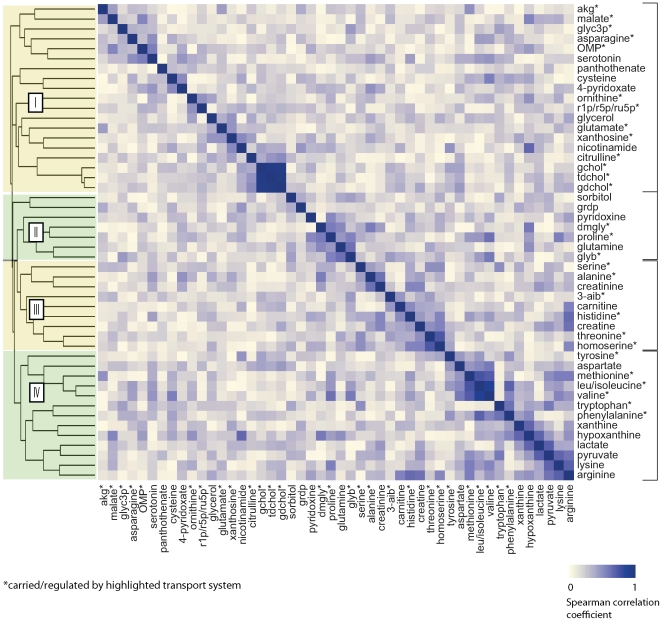
Hierarchical clustering of changed metabolites (FDR<0.05) in NGT Group. Grouping is according to 1−|ρ|, where ρ is the Spearman correlation coefficient for percentage change in metabolite abundance. Metabolite clusters that correspond to established transporter activities are highlighted. Cluster I corresponds to the SLC25A13 transporter (liver variant); Cluster II corresponds to SLC6A12; Cluster III corresponds to the small aliphatic system A transport system (SLC6, SLC7 and SLC38 transporters); and cluster IV corresponds to the hydrophobic/aliphatic system L transport system (SLC6, SLC7, SLC43).

**Figure 5:**
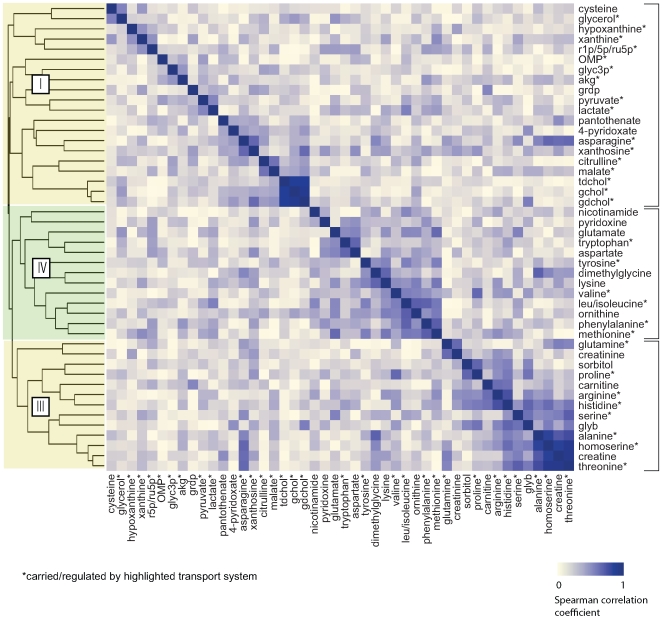
Hierarchical clustering of changed metabolites (FDR<0.05) in IGT Group. Grouping is according to 1−|ρ|, where ρ is the Spearman correlation coefficient for percentage change in metabolite abundance. Metabolite clusters that correspond to established transporter activities are highlighted. Cluster numbering is as in Figure 4.

Heatmaps of the results of hierarchical clustering (Figure 4,5) demonstrate that amino acids naturally group by transporter activity. For example, Clusters III and IV in NGT and IGT correspond to the activities of the System A and System L amino acid transporters, respectively. The System L transport activity is responsible for transporting large hydrophobic and aromatic amino acids with a particular preference for Phe, Leu/Ile (indistinguishable on our platform), Met, and Val. The corresponding peak intensities of these 4(+1) amino acids cluster tightly together across individuals (Spearman correlation coefficient 0.35–0.85 for NGT; 0.39–0.67 for IGT). Although Tyr and Trp show weaker correlation with the remaining system L amino acids, the clustering algorithm also groups them together in cluster IV. Likewise, 7 of the 10 (IGT) and 7 of the 12 (NGT) possible system A transport substrates, which are primarily small, neutral amino acids, are grouped into cluster III. The basic amino acids, which can be transported by System y+L (via exchange for the large hydrophobic amino acids), System A or System ATB,0+ carriers, show the most variability in terms of cluster membership.

Cluster II in NGT includes all 3 of the measured SLC6A12 substrates (proline, glycine betaine and dimethylglycine), which demonstrate absolute pairwise correlation coefficients ranging from 0.23 (for dimethylglycine and glycine betaine) to 0.57 (for glycine betaine and proline). Proline and glycine betaine also are strongly correlated and co-cluster in IGT (Spearman correlation coefficient = 0.60). The proline-betaine correlation likely reflects the fact that these metabolites are cotransported by at least three carriers (SLC6A12, SLC6A20 and SLC36A2). By contrast, these two metabolites are not known to participate in any common metabolic pathways, supporting the hypothesis that coordination of measured plasma levels of proline and glycine betaine is via regulation of their common transporters.

In Cluster I, bile salts are found with citrulline in both NGT and IGT. In IGT, malate also clusters closely with citrulline. We searched PubMed (http://www.ncbi.nlm.nih.gov/pubmed/) to find a connection between these metabolites and, interestingly, found that deficiency in the hepatic splice variant of the SLC25A13 protein (citrin), a component of the mitochondrial aspartate-malate shuttle involved in liver NAD+/NADH shuttling, leads to a buildup of both hepatic bile salts and citrulline [30]. SLC25A13 deficiency, caused by a large number of possible mutations, underlies two recessive Mendelian metabolic diseases: neonatal intrahepatic cholestasis (NICCD) characterized by liver bile salt accumulation and elevated citrulline plasma levels in infants and Type II Citrullinemia (CTLN2), which is characterized by elevated citrulline plasma levels in adults [31]. The widespread metabolic defects in the two diseases arise from a lack of cytoplasmic aspartate in the liver, an organ inherently limited in its ability to take up aspartate from plasma. Hepatic aspartate deficiency in turn leads to abnormalities in gluconeogenesis, ureagenesis, glycolysis, nucleotide biosynthesis, and triglyceride biosynthesis either because of the need for aspartate as a reactant or due to the resulting imbalance in the cytoplasmic NAD+/NADH ratio. Interestingly, an examination of Cluster I in NGT and IGT reveals that a large majority of changed metabolites correspond to pathways known to be regulated by liver aspartate levels and/or show abnormalities in SLC25A13 deficiency [32],[33]. These include pyrimidine biosynthesis (OMP, ribose-1-phosphate), purine biosynthesis (ribose-1-phosphate, xanthine, hypoxanthine, xanthosine), triglyceride biosynthesis (glycerol, glycerol-3-phosphate), urea cycle (citrulline, ornithine), bile salt accumulation (taurochenodeoxycholate, glycocholate, glycochenodeoxycholate), glycolysis (lactate, pyruvate), malate shuttling (malate, alpha-ketoglutarate), and aspartate biosynthesis (asparagine). The levels of several of these metabolites are known to be abnormal in affected humans and/or in mouse models of SLC25A13 deficiency [33]. Furthermore, in CTLN2, the abnormalities in these pathways are exacerbated by glucose intake [34], consistent with the observed OGTT-induced changes in metabolite levels.

**Figure 6:**
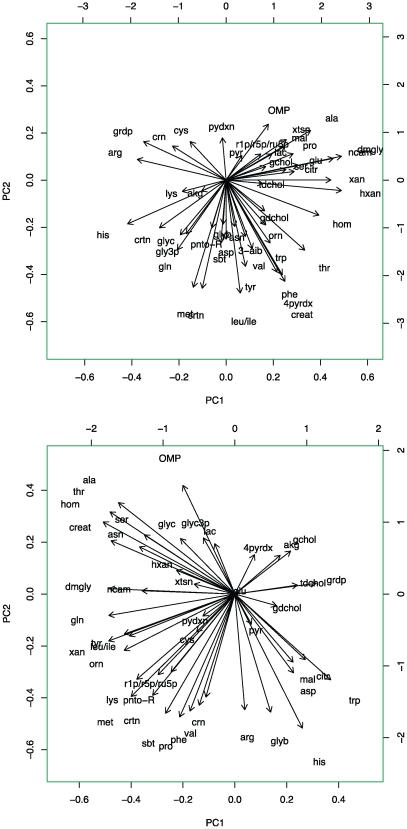
Principal component analysis of significantly changed metabolites (FDR<0.05) in NGT and IGT. Panels (a) and (b) correspond to NGT and IGT, respectively. Principal component #1 largely corresponds to pathways regulated by hepatic SLC25A13 activity, including glycolysis (lac, pyr) and gluconeogenesis (ala, ser), nucleotide biosynthesis (OMP, r1p, hxan, xan, xtsn, ncam), bile salt (gchol, tdchol) and citrulline (citr) accumulation, and NAD+/NADH balance by malate shuttling (glu, akg, mal). Principal component #2 largely corresponds to System A and L amino acid transport.

To further examine the relationship between distinct transport activities in OGTT metabolite profiling, we analyzed change in plasma levels of metabolites for NGT and IGT using principal component analysis (PCA). This analytic technique attempts to find linear combination of metabolites that best explain the interindividual variation seen in metabolite profiles. PCA revealed that the top two eigenvectors for NGT coincided with SLC25A13 and amino acid transport activities, respectively, explaining a total of 39% of interindividual variance in metabolite changes (see Figure 6). The discovery of orthogonal axes of variation corresponding to these known transport activities supports the importance of metabolite transport in OGTT profiles. The demarcation between the two types of transport was not as well seen for the top two IGT eigenvectors, which may reflect the significant heterogeneity in insulin resistance across the IGT group.

### Metabolite Transporters are Involved in Human Diseases

Given that metabolite profiling of perturbation experiments can interrogate specific underlying transporter activities, we investigated to what extent transporters are involved in human disease. We consulted the OMIM database of Mendelian diseases (http://www.ncbi.nlm.nih.gov/omim), and found 179 human disease phenotypes associated with transporter mutations. These include some of the transporters whose activity is reflected in OGTT metabolite profiles, such as SLC25A13, described above. In addition, the SLC6A14 amino acid/acyl-carnitine transporter, which primarily carries large hydrophobic and cationic amino acids, was both identified in our analysis as relevant to OGTT and previously been found to be associated with metabolic disease. Mutations in SLC6A14 have been shown to be associated with obesity in three independent populations [35],[36] and multiple SLC6A14 SNPs are suggestively associated (nominal *p*-value∼10^−4^–10^−5^) with waist circumference and weight in type 2 diabetes patients studied in the Diabetes Genomics Initiative genome-wide association study [37],[38]. Interestingly, a recent analysis of fasting metabolic profiles in obese, insulin-resistant patients revealed that a metabolic signature consisting of acyl-carnitine and branched chain and aromatic amino acids was highly correlated with obesity and insulin-resistance [39]. Although the authors attributed the relationship to dietary branch chain amino acid intake, we note the overlap with our glucose-stimulated System L transport cluster and with NGT principal component #2. Our results highlighting the importance of transporters in plasma metabolite profiles suggest that this signature may in part reflect basal insulin-responsive amino acid transporter activity.

### Limitations

Given that we have measured plasma levels for only a small fraction of the human metabolome, the pathway models that we have discovered may be smaller than the actual enriched pathway. Conversely, for those AMGs that include unmeasured metabolites, measurement of additional metabolites may show that other pathways more convincingly explain the observed physiological changes. A further limitation is that our scoring method considered all significant changes in plasma levels equivalently, without considering direction of change. Additionally, we expect there are alterations in metabolic reaction flux within the cell in response to glucose challenge that may be difficult to decipher from plasma metabolite levels. Finally, metabolites that are significantly changed but which are not closely linked to other metabolites via chemical reactions or shared enzymes/transporters are unlikely to appear in AMGs, but may still reflect important altered tissue activities during OGTT.

## Discussion

We have directly integrated metabolic reaction connectivity and a collection of shared transporter relationships, extended here by manual literature curation, into metabolite profile interpretation to identify biologic processes relevant to a physiological perturbation experiment. Our approach makes use of a deterministic approach to identify active modules and directly integrates plasma measurements of metabolites with a unipartite graph capturing interrelationship between metabolic substrates. Through this method, we have uncovered a potentially important contribution for transporter activities in plasma metabolite profiles, which is ignored by using more traditional analysis of metabolic pathways.

A prior application of active modules to metabolism relied on integrating microarray-derived gene expression information for enzymes with enzyme connectivity in metabolic graphs to identify clusters of functionally connected enzymes that collectively show a high degree of change in a perturbation experiment [18]. In the same study, “reporter metabolites” were identified by connection to enzymes that show a high degree of change at a transcriptional level. Our method allows deriving potentially unexpected underlying pathways from direct metabolite measurements, which should prove increasingly valuable given the emergence of metabolic profiling in both biological and clinical arenas [40].

One motivation for this approach is to redefine human disease in terms of aberrant metabolic activities. Given groups of affected and control individuals, active module analysis can achieve this in a number of ways. Baseline differences in each metabolite's abundance can be scored between affected and unaffected individuals [39] and interpreted in the context of a metabolic reaction network. As opposed to the lists of significantly changed biomarkers commonly generated in association analyses, the data integration involved in active module analysis allows hypotheses generation in terms of more meaningful biological activities. As an alternative to baseline comparisons, perturbation experiments such as OGTT (or medication or exercise) can identify physiologic pathways associated with the perturbation by identifying active modules within the control group. In this way, analysis of differences between affected and control individuals can be more tightly focused on metabolites relevant to the normal physiological process. Our study used this approach to suggest differences in particular transporter and enzyme activities between the NGT and IGT groups in response to glucose challenge. Using a perturbation experiment, differences in the induced change in metabolite abundance between affected and unaffected patients can also be scored directly and mapped onto a metabolic reaction graph to identify differentially active modules. Such modules may capture distinct facets of a heterogeneous disease. Finally, longitudinal data for a group of affected individuals permits subclassification of disease via active module-based metabolite scores that measure the ability to predict some adverse outcome (for example heart attacks in individuals with stable coronary artery disease or diabetes development in individuals with impaired glucose tolerance). A similar approach used gene expression profiles [8] in the context of a protein-protein interaction network to predict the likelihood of cancer metastasis within individuals with breast cancer.

As the relationship of metabolite abundance with disease incidence and outcomes is better understood, we may ultimately be able to use integrated analyses of metabolic profiles to subclassify disease on the basis of distinct enzymatic/transporter activities, thus allowing a more individualized approach to clinical medicine.

## Methods

### Physiologic Perturbation Experiments

Metabolite abundance measurements from an oral glucose tolerance test have been described [10]. Briefly, 50 individuals from the Framingham Offspring Study, 25 with normal fasting glucose and normal glucose tolerance (OGTT-NGT) and 25 with impaired glucose tolerance (OGTT-IGT) were selected for metabolic profiling. The Framingham Heart Study is a longitudinal community-based investigation that was initiated in 1948 to prospectively identify cardiovascular disease risk factors [41]. The children and their spouses of the original cohort were recruited in 1971 and constitute the Framingham Offspring Study Cohort. All subjects were white and of European descent. OGTT was administered routinely at the baseline exam. After a 12-hour overnight fast participants were given 75 g glucose in solution orally. Blood samples were drawn fasting and 120 minutes after glucose ingestion and after HPLC purification, metabolite abundances were determined using a triple quadrupole mass spectrometer as previously described [10].

### Mass Spectrometry Analysis

Metabolite peak intensities were determined as described previously [10]. We eliminated metabolites from subsequent analysis that: 1) were not included in the Recon 1 network; 2) were confounded by a potential contaminant; or 3) were measured in <50% of individuals In cases where two or more metabolites could not be separated on the basis of chromatographic elution patterns and parent and daughter mass-to-charge ion spectra, peak intensities for that group of metabolites were randomly assigned to one of the possible metabolites during each of the 100 simulations (see below). This ambiguity was only present for 3 groups of metabolites (leucine/isoleucine; ribose-5-phosphate/ribose-1-phosphate/ribulose-5-phosphate, citrate/isocitrate), with members of each group showing chemical similarity and participating in many common reactions in Recon 1. By these criteria, the initial list of 171 peaks measured on the platform was thus narrowed to abundances for 88 and 84 metabolites, respectively, for the NGT and IGT groups. A one-sample Wilcoxon signed rank test was used to determine the statistical significance for change for the metabolite peak intensities for both groups (percent difference in peak magnitude after glucose challenge relative to baseline).

### Signal-Noise Decompositon

In order to identify significantly changed clusters of functionally connected metabolites, we converted experimental *p*-values from the Wilcoxon test to scores. Although a variety of methods can be selected for this purpose, we used the beta-uniform distribution method [12],[42], which allows control of false-positive and false-negative rates in the analysis. For NGT and IGT separately, the distribution of *p*-values was modeled as a mixture of a beta distribution for the signal and a uniform distribution for the noise as shown below:
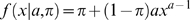
(1)where *x* is a given *p-*value, f(*x*) is the probability density at *x*, *π* represents the mixture parameter, and *a* represents a shape parameter for the beta distribution. The values of *π* and *a* were fit using the *optim* function in R. The parameter *τ* was chosen to achieve an FDR of 0.01. *p*-values were converted into scores as described in [12],[42], where the score for each metabolite at the chosen FDR, S_FDR_(x), is computed as follows:
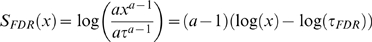
(2)


Using this statistic, nodes for which the *p*-value>*τ*, will have a negative score.

### Construction of Metabolic Reaction Network

A Metabolic Reaction Network (MRN) was constructed based on the 3338 metabolic reactions in Recon 1. We treated all 1500 reactant and product metabolites in Recon 1 as nodes. Cellular locations were assigned to each metabolite as specified in Recon 1, and metabolites were split into multiple nodes (each corresponding to a different location). This process resulted in 2779 total nodes.

For each metabolic reaction, edges in the MRN were drawn between each reactant and product nodes and between all common metabolites substrates (reactants and products) of enzymes and transporters. Furthermore, since transporter annotation was not complete, for each of the measured metabolites we manually searched the literature to identify transporter-substrate relationships and identified all substrates for any transporter found. Finally, we expanded the list of reactions so that each transporter-reaction relationship was reflected in an independent entry. This approach resulted in an additional 737 reactions (see Table S2).

To improve the specificity of the active module discovery process, nodes and edges involving the following 28 ‘promiscuous’ and/or buffered metabolites were also eliminated: UMP, UDP, UTP, FAD, FADH_2_, Na^+^, K+, SO_4_, NH_4_, CO_2_, Phosphate, O_2_, Pyrophosphate, H_2_O, H^+^, OH^−^, ATP, ADP, AMP, CTP, CDP, CMP, NAD, NADH, NADP, NADPH, H_2_O_2_, and HCO3^−^. As many of these metabolites serve as cofactors, their inclusion would contribute to non-specific bridges between metabolites in active modules and would thus be of limited use for biological processes. We did however include nodes and edges for reactions where nucleotides serve as primary reactants and products, such as those involved in nucleotide biosynthesis or catabolism; NAD metabolism; and Riboflavin metabolism.

Because abundance measurements were only available for a small fraction of the metabolic network, we limited the MRN to the union of measured metabolites and all nodes found on paths (up to path-length three) between two measured nodes. This filtering, used to reduce computing cost, did not alter downstream results because to be included in an active module, a metabolite must either be measured or lie on a path between measured metabolites.

Scores generated above were assigned to measured nodes in the MRN. We built both a Scored Extracellular MRN (EMRN) and a separate Scored Cytoplasmic MRN (CMRN). For the EMRN, if a metabolite had two cellular locations, the metabolite score was assigned to the extracellular metabolite, modeling extracellular levels; for the CMRN, scores, in such a situation, were assigned to the cytoplasmic metabolite. The EMRN and CMRN networks had 297 nodes and 5515 edges and 344 nodes and 6089 edges, respectively, after applying the above steps.

### Determination of Active Subnetworks

To identify active modules in our MRN, we used a recently developed method [12] that provides an exact solution to the problem of finding a group of connected nodes with the highest combined score by transforming the problem to that of the Prize-Collecting Steiner Tree. The method requires that each metabolite in the network have a score (see above for details on the required properties of that score). Because we had scores assigned for only a fraction of the network nodes, *p-*values for unmeasured nodes were sampled randomly from the uniform distribution expected of unchanged metabolites, thus sampling the joint distribution of unmeasured metabolite abundance under the null hypothesis. Such an approach is common practice in Bayesian data analysis to estimate posterior probability distributions and accounts for our uncertainty about unmeasured metabolites more accurately than would asserting that we are sure that the metabolite has not changed. It also permits an unmeasured metabolite to be included in a cluster if there is sufficient support, in that the unmeasured metabolite bridges multiple high-scoring measured metabolites. Scores were then determined for the unmeasured nodes as described in Methods, with *π*, *a*, and *τ* determined using the distribution of measured *p*-values. We repeated the random sampling 100 times for each scored network and identified one optimal solution per network. We explored searching also for suboptimal solutions but found that these primarily consisted of a subset of the metabolites of the optimal solution rather than other distinct areas of the graph.

### Statistical Significance of Active Subnetwork Solutions

For the purpose of evaluating statistical significance of observed active modules, we generated random solutions by repeating the active module discovery process for 100 random scored MRNs. Although the topology was preserved, the scores for each random MRN were randomly permuted among measured nodes and *p*-values for unmeasured nodes were sampled uniformly at random. The distribution of scores for the original and permuted data were compared by a one-sided Mann-Whitney-Wilcoxon test using the *R* package (www.Rproject.org).

### Predefined Pathway and Enzyme/Transporter Enrichment of Active Module Clusters

For each scored MRN, the frequency of appearance in the corresponding 100 solutions was measured for all nodes. Nodes with ≥0.20 relative frequency were grouped together to form an Active Module Group (AMG), which was examined for significant overlap with metabolite sets corresponding to predefined pathways. To identify predefined pathway enrichment in the AMGs, we used a modified version of the FuncAssociate program [14]. FuncAssociate takes as input a query list of metabolites and a mapping of pathways to metabolites. For each pathway, it tests for enrichment of that pathway in the query list relative to the “tested universe” of metabolites by using the cumulative hypergeometric (Fisher's Exact) test. Adjustment for multiple hypothesis testing is achieved by resampling [14]. Briefly, the null distribution of *p*-values is generated by repeating the test for 1000 randomly generated query lists of the same size from the same universe of metabolites. For each random query list, the minimum *p*-value observed for any pathway is retained. The adjusted *p*-value (*p*_adj_) is then the fraction of random query lists that yield a minimum *p*-value equal to or lower than the minimum *p*-value observed.

We generated pathway and enzyme/transporter-to-metabolite mappings for Recon 1, limiting our analysis to pathways, enzymes or transporters that included at least three metabolites in the metabolite universe. We were interested in enrichment for analysis for both AMGs and for our ranked list of changed metabolite. To look for pathway enrichment in the ranked list of changed metabolites, we used the “ordered” setting in FuncAssociate (http://llama.med.harvard.edu/cgi/func1/ funcassociate_advanced), which tests a ranked list of metabolites and finds the rank cutoff that optimizes significance (using resampling to adjust for multiple testing). For this analysis, the metabolite list was ranked in both increasing and decreasing order of magnitude of change.

For assessing pathway and enzyme/transporter enrichment in our AMGs, we used as our universe of metabolites all nodes in the reduced networks. To address the fact that the AMGs may be biased in composition towards measured nodes, we modified FuncAssociate so that the null distribution of *p*-values was obtained using randomly generated metabolite lists that matched the query list in composition of measured and unmeasured nodes. We repeated the same process for enzymes and transporters, generating mappings for all Recon 1 reactions for which an enzyme or transporter could be identified.

### Tissue of Origin Enrichment of Active Module Clusters

In a recent manuscript [19], tissue-specific metabolic fluxes were predicted for the Recon 1 network. The authors solved a constraint-based modeling optimization problem by finding a metabolic flux distribution that satisfied constraints imposed by stoichiometric and thermodynamic conditions of the network and maximized agreement between flux and enzyme mRNA and protein expression for ten human tissues. We used the authors' predictions for metabolite activity in each of the ten tissues and looked for tissue enrichment for our measured metabolites and active modules clusters. For each metabolite and cellular location within each tissue, the authors provided an activity score that ranged from −2 to +2, where positive scores indicated activity, negative scored indicated inactivity and the magnitude of the score indicated confidence in the prediction. A score of 0 indicated an ambiguous activity level. For each AMG, we computed an activity score for each tissue by summing the individual scores for each metabolite. The null distribution of scores was obtained as above, where 1000 random sets of metabolites were selected from the node universe, matching the AMG in composition of measured and unmeasured nodes, and the highest tissue score taken for each set.

### False Discovery Rate, Hierarchical Clustering and Principal Component Analysis of Changed Metabolites

Hierarchical clustering and PCA was performed on the subset of metabolites that changed significantly in either NGT or IGT at an FDR<0.05, determined using the *qvalue* package in R (www.Rproject.org). L-Alanine and L-Cysteine, which were the only measured standard amino acids that failed to change significantly in either group, were also included. Input data corresponded to fractional change in metabolite abundance across individuals. For clustering, the absolute value of the Spearman coefficient was used to compute the dissimilarity matrix. Heatmaps and principal component analyses were performed using the *heatmap* and *prcomp* functions, respectively, in the *R* package.

### Sensitivity of Active Module Results to Alterations in Parameter Values/Thresholds

In our analysis, three parameters determined the balance between measured and unmeasured metabolites in our active module solutions: 1) the false-discovery rate (FDR) threshold used in the determination of scores for measured metabolites; 2) the path-length threshold between measured metabolites used in filtering unmeasured metabolites from the MRN; and 3) the frequency threshold used in selecting active module solution metabolites for inclusion into active module groups. Although unmeasured metabolites are useful for hypothesis generation in terms of identifying potentially novel markers of insulin function, such hypotheses should not be so numerous as to dominate the analysis. At the stringent FDR of 0.01 selected, the active module discovery process was relatively insensitive to changes in the other two parameters, with little difference in the observed results at higher or lower path lengths. In fact, all unmeasured metabolites in active module groups were directly connected to one or more measured metabolites. For the frequency cutoff for active module group metabolites, our primary conclusions were robust to all thresholds from 0.10 to 0.50.

## Supporting Information

Table S1 Metabolite profiles for NGT and IGT groups. Recon 1 symbols and names for measured metabolites are listed alongside magnitude (fraction of baseline) and significance (*p*-value) of change in abundance after glucose challenge. Metabolite abundances pre- and post- glucose challenge are also provided in separate tabs for the NGT and IGT groups.
(0.13 MB XLS)

Table S2 Manually curated transport reactions used in addition to Recon 1 reactions to derive Metabolite Reaction networks. The first tab corresponds to 190 reactions already described in Recon 1 - a column was added that makes explicit the mapping to a single solute transporter. The second tab corresponds to 737 additional transport reactions identified by manual literature curation - these have also each been mapped to a single transporter.
(0.22 MB XLS)

Table S3 Relative frequencies for metabolites in Active Module Solutions. A frequency threshold of 0.20 for appearance of metabolites was used for subsequent analysis and figure construction.
(0.07 MB XLS)

Table S4 Metabolic reactions and/or enzymes/transporters corresponding to edges in AMGs. Edges correspond to reactant-product pairs in individual reactions and/or substrates of common enzymes/transporters.
(0.12 MB XLS)

Figure S1 Active Module Group from a) IGT-EMRN and b) IGT-CMRN. For details see legend for Figure 2.
(0.46 MB EPS)

## References

[1] Loscalzo J, Kohane I, Barabasi AL (2007). Human disease classification in the postgenomic era: a complex systems approach to human pathobiology.. Mol Syst Biol.

[2] Zheng SL, Sun J, Wiklund F, Smith S, Stattin P (2008). Cumulative association of five genetic variants with prostate cancer.. N Engl J Med.

[3] Bild AH, Yao G, Chang JT, Wang Q, Potti A (2006). Oncogenic pathway signatures in human cancers as a guide to targeted therapies.. Nature.

[4] Kon OL, Yip TT, Ho MF, Chan WH, Wong WK (2008). The distinctive gastric fluid proteome in gastric cancer reveals a multi-biomarker diagnostic profile.. BMC Med Genomics.

[5] Golub TR, Slonim DK, Tamayo P, Huard C, Gaasenbeek M (1999). Molecular classification of cancer: class discovery and class prediction by gene expression monitoring.. Science.

[6] Acharya CR, Hsu DS, Anders CK, Anguiano A, Salter KH (2008). Gene expression signatures, clinicopathological features, and individualized therapy in breast cancer.. JAMA.

[7] Nicholson JK, Holmes E, Lindon JC, Wilson ID (2004). The challenges of modeling mammalian biocomplexity.. Nat Biotechnol.

[8] Chuang HY, Lee E, Liu YT, Lee D, Ideker T (2007). Network-based classification of breast cancer metastasis.. Mol Syst Biol.

[9] Lee E, Chuang HY, Kim JW, Ideker T, Lee D (2008). Inferring pathway activity toward precise disease classification.. PLoS Comput Biol.

[10] Shaham O, Wei R, Wang TJ, Ricciardi C, Lewis GD (2008). Metabolic profiling of the human response to a glucose challenge reveals distinct axes of insulin sensitivity.. Mol Syst Biol.

[11] Duarte NC, Becker SA, Jamshidi N, Thiele I, Mo ML (2007). Global reconstruction of the human metabolic network based on genomic and bibliomic data.. Proc Natl Acad Sci U S A.

[12] Dittrich MT, Klau GW, Rosenwald A, Dandekar T, Muller T (2008). Identifying functional modules in protein-protein interaction networks: an integrated exact approach.. Bioinformatics.

[13] Ideker T, Ozier O, Schwikowski B, Siegel AF (2002). Discovering regulatory and signalling circuits in molecular interaction networks.. Bioinformatics.

[14] Berriz GF, King OD, Bryant B, Sander C, Roth FP (2003). Characterizing gene sets with FuncAssociate.. Bioinformatics.

[15] Rossell S, van der Weijden CC, Lindenbergh A, van Tuijl A, Francke C (2006). Unraveling the complexity of flux regulation: a new method demonstrated for nutrient starvation in Saccharomyces cerevisiae.. Proc Natl Acad Sci U S A.

[16] Reiss DJ, Avila-Campillo I, Thorsson V, Schwikowski B, Galitski T (2005). Tools enabling the elucidation of molecular pathways active in human disease: application to Hepatitis C virus infection.. BMC Bioinformatics.

[17] Bandyopadhyay S, Kelley R, Ideker T (2006). Discovering regulated networks during HIV-1 latency and reactivation.. Pac Symp Biocomput.

[18] Patil KR, Nielsen J (2005). Uncovering transcriptional regulation of metabolism by using metabolic network topology.. Proc Natl Acad Sci U S A.

[19] Shlomi T, Cabili MN, Herrgard MJ, Palsson BO, Ruppin E (2008). Network-based prediction of human tissue-specific metabolism.. Nat Biotechnol.

[20] Kitamura T, Kahn CR, Accili D (2003). Insulin receptor knockout mice.. Annu Rev Physiol.

[21] Skott P, Hother-Nielsen O, Bruun NE, Giese J, Nielsen MD (1989). Effects of insulin on kidney function and sodium excretion in healthy subjects.. Diabetologia.

[22] Broer S (2008). Amino acid transport across mammalian intestinal and renal epithelia.. Physiol Rev.

[23] Hatanaka T, Hatanaka Y, Tsuchida J, Ganapathy V, Setou M (2006). Amino acid transporter ATA2 is stored at the trans-Golgi network and released by insulin stimulus in adipocytes.. J Biol Chem.

[24] Hyde R, Cwiklinski EL, MacAulay K, Taylor PM, Hundal HS (2007). Distinct sensor pathways in the hierarchical control of SNAT2, a putative amino acid transceptor, by amino acid availability.. J Biol Chem.

[25] Roos S, Lagerlof O, Wennergren M, Powell T, Jansson T (2009). Regulation of amino acid transporters by glucose and growth factors in cultured primary human trophoblast cells is mediated by mTOR signaling.. Am J Physiol Cell Physiol.

[26] Wang W, Fridman A, Blackledge W, Connelly S, Wilson IA (2009). The phosphatidylinositol 3-kinase/akt cassette regulates purine nucleotide synthesis.. J Biol Chem.

[27] Chen NH, Reith ME, Quick MW (2004). Synaptic uptake and beyond: the sodium- and chloride-dependent neurotransmitter transporter family SLC6.. Pflugers Arch.

[28] Kempson SA, Montrose MH (2004). Osmotic regulation of renal betaine transport: transcription and beyond.. Pflugers Arch.

[29] Matskevitch I, Wagner CA, Stegen C, Broer S, Noll B (1999). Functional characterization of the Betaine/gamma-aminobutyric acid transporter BGT-1 expressed in Xenopus oocytes.. J Biol Chem.

[30] Palmieri F (2004). The mitochondrial transporter family (SLC25): physiological and pathological implications.. Pflugers Arch.

[31] Saheki T, Kobayashi K (2002). Mitochondrial aspartate glutamate carrier (citrin) deficiency as the cause of adult-onset type II citrullinemia (CTLN2) and idiopathic neonatal hepatitis (NICCD).. J Hum Genet.

[32] Yeh JN, Jeng YM, Chen HL, Ni YH, Hwu WL (2006). Hepatic steatosis and neonatal intrahepatic cholestasis caused by citrin deficiency (NICCD) in Taiwanese infants.. J Pediatr.

[33] Saheki T, Iijima M, Li MX, Kobayashi K, Horiuchi M (2007). Citrin/mitochondrial glycerol-3-phosphate dehydrogenase double knock-out mice recapitulate features of human citrin deficiency.. J Biol Chem.

[34] Saheki T, Kobayashi K, Terashi M, Ohura T, Yanagawa Y (2008). Reduced carbohydrate intake in citrin-deficient subjects.. J Inherit Metab Dis.

[35] Durand E, Boutin P, Meyre D, Charles MA, Clement K (2004). Polymorphisms in the amino acid transporter solute carrier family 6 (neurotransmitter transporter) member 14 gene contribute to polygenic obesity in French Caucasians.. Diabetes.

[36] Suviolahti E, Oksanen LJ, Ohman M, Cantor RM, Ridderstrale M (2003). The SLC6A14 gene shows evidence of association with obesity.. J Clin Invest.

[37] Saxena R, Voight BF, Lyssenko V, Burtt NP, de Bakker PI (2007). Genome-wide association analysis identifies loci for type 2 diabetes and triglyceride levels.. Science.

[38] Johnson AD, O'Donnell CJ (2009). An open access database of genome-wide association results.. BMC Med Genet.

[39] Newgard CB, An J, Bain JR, Muehlbauer MJ, Stevens RD (2009). A branched-chain amino acid-related metabolic signature that differentiates obese and lean humans and contributes to insulin resistance.. Cell Metab.

[40] Sreekumar A, Poisson LM, Rajendiran TM, Khan AP, Cao Q (2009). Metabolomic profiles delineate potential role for sarcosine in prostate cancer progression.. Nature.

[41] Kannel WB, Feinleib M, McNamara PM, Garrison RJ, Castelli WP (1979). An investigation of coronary heart disease in families. The Framingham offspring study.. Am J Epidemiol.

[42] Pounds S, Morris SW (2003). Estimating the occurrence of false positives and false negatives in microarray studies by approximating and partitioning the empirical distribution of p-values.. Bioinformatics.

